# Sucrose non-ferment 1 related protein kinase 2 (*SnRK*2) genes could mediate the stress responses in potato (*Solanum tuberosum* L.)

**DOI:** 10.1186/s12863-017-0506-6

**Published:** 2017-05-15

**Authors:** Jiangping Bai, Juan Mao, Hongyu Yang, Awais Khan, Aqi Fan, Siyan Liu, Junlian Zhang, Di Wang, Huijuan Gao, Jinlin Zhang

**Affiliations:** 1 Gansu Key Lab of Crop Improvement & Germplasm Enhancement, Gansu Provincial Key Lab of Aridland Crop Science, Lanzhou, 730070 Gansu People’s Republic of China; 2 College of Agronomy, Gansu Agricultural University, Lanzhou, 730070 Gansu People’s Republic of China; 3 College of Horticulture, Gansu Agricultural University, Lanzhou, 730070 Gansu People’s Republic of China; 4 International Potato Center (CIP), Avenida La Molina 1895, La Molina Apartado, 1558 Lima, Peru; 5 State Key Laboratory of Grassland Agro-ecosystems, College of Pastoral Agriculture Science and Technology, Lanzhou University, Lanzhou, 730020 Gansu People’s Republic of China

**Keywords:** Potato, *StSnRK2*, Stress responses, ABA, NaCl, PEG-6000

## Abstract

**Background:**

The SnRKs (sucrose non-fermenting 1 related protein kinase) are a gene family coding for Ser/Thr protein kinases and play important roles in linking the tolerance and metabolic responses of plants to abiotic stresses. To date, no genome-wide characterization of the sucrose non-ferment 1 related protein kinase 2 (SnRK2) subfamily has been conducted in potato (*Solanum tuberosum* L.).

**Results:**

In this study, eight *StSnRK*2 genes (*StSnRK*2.1- *StSnRK*2.8) were identified in the genome of the potato (*Solanum tuberosum* L.) cultivar ‘*Longshu* 3’, with similar characteristics to *SnRK*2 from other plant species in gene structure, motif distribution and secondary structures. The C-terminal regions were highly divergent among StSnRK2s, while they all carried the similar Ser/Thr protein kinase domain. The fluorescence of GFP fused with StSnRK2.1, StSnRK2.2, StSnRK2.6, StSnRK2.7 and StSnRK2.8 was detected in the nucleus and cytoplasm of onion epidermal cells with StSnRK2.3 and StSnRK2.4 mainly associated to the nucleus while StSnRK2.5 to subcellular organelles. Expression level analysis by qRT-PCR showed that *StSnRK*2.1, 2.2, 2.5 and 2.6 were more than 1 fold higher in the root than in the leaf, tuber and stem tissues. The expressions of *StSnRK*2.3, 2.7, and 2.8 were at least 1.5 folds higher in the leaf and stem than in the root, but lower in the tuber. The expression of *StSnRK*2.4 was also significantly (*P* < 0.05) higher in leaf, stem, and tuber than in the root. From the perspective of the relative expressions of *StSnRK2* genes in potato, ABA treatment had a different effect from NaCl and PEG treatments.

**Conclusion:**

In the present study, we identified and characterized eight *SnRK2s* in the potato genome. The eight *StSnRK2s* exhibit similar gene structure and secondary structures in potato to the *SnRK2s* found in other plant species. The relative expression of eight genes varied among various tissues (roots, leaves, tubers, and stems) and abiotic stresses (ABA, NaCl and PEG-6000) with the prolongation of treatments. This study provides valuable information for the future functional dissection of potato *SnRK2* genes in stress signal transduction, plant growth and development.

**Electronic supplementary material:**

The online version of this article (doi:10.1186/s12863-017-0506-6) contains supplementary material, which is available to authorized users.

## Background

In many regions of the world, potato (*Solanum tuberosum* L.) production is seriously threatened by abiotic stresses such as frequent drought and salinity [1]. Plant drought tolerance is a complex response and involves the comprehensive interactions of numerous genes, proteins and metabolites in plant cells. Systematic analysis of the plant cell network responsible for drought stress tolerance is a promising approach for the development of drought tolerant plants [1, 2]. Protein phosphorylation is involved in regulation of various cellular activities in plants and one of the main signals mediating the responses to environmental stresses [3–7]. The SnRKs (sucrose non-fermenting 1 related protein kinase) are a gene family coding for Ser/Thr protein kinases and play important roles in linking abiotic stress tolerance and the metabolic responses of plants [8–10]. Based on sequence similarity, domain structure and metabolic roles, the plant SnRK family is divided into three subfamilies: SnRK1, SnRK2 and SnRK3. Many studies have demonstrated that these three subfamilies play various roles in the metabolism and development of plants. SnRK1 plays an important role in regulating carbon metabolism and energy conversion in plants [11, 12], while SnRK3 is involved in plant development, calcium-responsive regulatory loop and abscisic acid (ABA) sensitivity. SnRK2 members are the major players in plant responses to osmotic stresses [13–16], ABA dependent and independent stomatal closure-opening [17], fruit development [18], seed dormancy [19] and germination [20, 21].

Since the first *SnRK2* (PKABA1) gene was identified in wheat [22], the members of the *SnRK2* subfamily have been subsequently identified in many other plant species such as *Arabidopsis* [23, 24], rice [25], maize [26], tobacco [27], wheat [28, 29], sorghum [30], soybean [31, 32], barley [33] and grape [34]. In *Arabidopsis*, nine of the ten *SnRK2* members (except for *SnRK2.9*) can be activated by osmotic stress [13]. Among them, *SnRK2.8* and *SnRK2.7* play a critical role in regulating the expression of drought-responsive genes [35]. In rice, all the ten *SnRK2* members (designated as *SAPK1* to *SAPK10*, stress/ABA-activated protein kinase) are activated by hyperosmotic stress with three of them (*SAPK8*, *SAPK9*, and *SAPK10*) also activated by ABA [25, 36]. In maize, all the ten characterized members of *SnRK2* are stress-related [26]. In wheat, osmotic stress and ABA-induced gene expression were linked with the activity of SnRK2 members [28, 29]. However, little is known about the functions of *SnRK2s* in potato and there is no information on how *SnRK2* family genes are involved in the tolerance of potato to osmotic stress.

In this study, we identified and characterized eight *SnRK2* genes from the potato genome (named *StSnRK2.1, StSnRK2.2, StSnRK2.3, StSnRK2.4, StSnRK2.5, StSnRK2.6, StSnRK2.7* and *StSnRK2.8*), then analyzed their tissue-specific and stress-induced expression profiles. To better understand their mechanisms underlying improved abiotic stress tolerance of the *StSnRK2* gene family, the subcellular localization of StSnRK2 proteins, the expression patterns of the eight *SnRK2s* gene members and physiological index analysis of potato plantlets responding to ABA (50 μM), NaCl (200 mM) and PEG-6000 (5%) were performed. This study established functions for the potato *SnRK2* gene family and provides a foundation for further clarifying the mechanism of potato stress resistance. The results will also provide genetic foundations for developing drought tolerant potato cultivars via manipulating *SnRK2* gene family.

## Methods

### Plant material

One the local main potato cultivars, ‘Longshu 3’ released by Gansu Academy of Agricultural Sciences, Lanzhou, China, was used in the study. This cultivar is widely grown in northwestern China because of its moderate resistance to low temperatures, drought and salinity. Potato plantlets were propagated in MS medium [37]. A total of 6–8 plantlets were cultured in each 150 ml flask at an illumination intensity of 200 μmol m^2^ s^−1^ under the temperature of 23 ± 2 °C with a photoperiod of 16 h/8 h (day/night).

### Identification and cloning of potato StSnRK2s

The coding sequences of *SnRK2s* from *Arabidopsis*, maize, and rice were obtained through literature and the genbank (http://www.ncbi.nlm.nih.gov) (Additional file 1: Table S1). To identify the *SnRK2* genes in potato, we looked for the highly conserved sequences of *SnRK2s* in the Potato Genome Sequencing Consortium database (http://potatogenome.net/index.php/Main_Page).

To clone the cDNA sequences, total RNA was extracted from the potato plantlets using the RNA simple Total RNA Kit (TIANGEN). The quality and quantity of RNA were measured by both electrophoresis and optical absorbency (Nanophotometer, Implen). The cDNA was synthesized from the total RNA following the instruction of the Kit cDNA MMLV (Sangon). Full-length coding sequences of *StSnRK2* genes were amplified via PCR with the primers presented in Additional file 1: Table S2. The PCR products were inserted into pGEM-T easy cloning vector and propagated in *Escherichia coli* DH5α (Promega) for sequencing and conservation.

### Phylogenetic analysis

The *SnRK*2*s* from *Arabidopsis*, rice, maize and potato were aligned using ClustalX1.81 (ftp://ftp-igbmc.u-strasbg.fr/pub/ClustalX). The final view of the alignment was manually adjusted with Jalview (2.07) [38]. The phylogenetic tree was constructed using the neighbor-joining (NJ) method in MEGA 5 [39]. Bootstrap analysis was performed using 1000 replicates to evaluate the reliability of different phylogenetic groups.

### Characterization of StSnRK2 genes ﻿and proteins﻿

The genomic sequences were identified from Potato Genome Sequencing Consortium database (http://potatogenome.net/index.php/Main_Page). The gene structures (exon and intron) were analyzed with splign (http://www.ncbi.nlm.nih.gov/sutils/splign) [40]. The promoter sequences were identified about 2 kb upstream of the transcription start site of each gene, and the abiotic stress-associated elements (ABRE, DRE/CRT, and LTRE) were obtained from the PLACE database (http://www.dna.affrc.go.jp/) [41]. The secondary structure of the deduced polypeptide was predicted using the programs of SOPMA which listed in Expasy (www.EXPASY.org)

### Subcellular localization of StSnRK2s

To determine the subcellular localization of the StSnRK2s, full-length of *StSnRK2s* were inserted into the KpnI/BamHI site of the pEBGFP vector to generate pEBGFP-StSnRK2. For transient expression in onion epidermal cells, the pEBGFP and pEBGFP-StSnRK2s plasmids and the constructs were introduced into onion epidermal cells using *Agrobacterium tumefaciens*-mediated transformation methods. Onion epidermal tissues were subsequently incubated on solid MS medium in the dark at 26 °C for 48 h. Localization of fluorescent proteins in onion epidermal cells was observed by a Zeiss LSM 710/ConfoCor2 laser-scanning imaging system (CarlZeiss, Jena, Germany). Fluorescence was detected between 505 and 550 nm with excitation at 488 nm. GFP fluorescence and light field vision were recorded in separate channels and then merged into an overlay image.

### Expression profile of StSnRK2s in potato plants

Four-week old potato in vitro plantlets were transferred into liquid MS medium supplemented with either 50 μM ABA, 200 mM sodium chloride (NaCl) or 5% polyethyleneglycol (PEG-6000) to investigate the expression patterns of StSnRK2s under different stress-conditions. After incubation 24 h, the leaves were harvested and immediately frozen in liquid nitrogen for RNA extraction. Quantitative Real-Time PCR (qRT-PCR) was conducted with SYBR Green I technology (TaKaRa), the ef1α gene (AB061263) was used as internal control gene, the gene primers presented in Additional file 1: Table S2 and the relative quantification of RNA expression was selected. Three independent replicates were performed for each experiment. In each case, ten test tubes containing six to eight explants represented each treatment. For quantification, a no-template control, calibration curve and non-specific reactions were run in triplicate. The 2^-△△Ct^ method (C_t_, Cycle threshold value of target gene) was selected to calculate the gene expression folds; The formulas are as follows [42, 43]:

$$ \triangle {\mathrm{C}}_{\mathrm{t}\ \mathrm{Test}\ \mathrm{gene}}={\mathrm{C}}_{\mathrm{t}\ \mathrm{Test}\ \mathrm{gene}}-{\mathrm{C}}_{\mathrm{t}\ \mathrm{Housekeeping}\ \mathrm{gene}} $$

$$ \triangle {\mathrm{C}}_{\mathrm{t}\ \mathrm{Control}\ \mathrm{gene}}={\mathrm{C}}_{\mathrm{t}\ \mathrm{Control}\ \mathrm{gene}}-{\mathrm{C}}_{\mathrm{t}\ \mathrm{Housekeeping}\ \mathrm{gene}}, $$

where control gene refers to those genes that were treated with only water.

$$ -\triangle \triangle {\mathrm{C}}_{\mathrm{t}}=-\left(\triangle {\mathrm{C}}_{\mathrm{t}\ \mathrm{Test}\ \mathrm{gene}}-\triangle {\mathrm{C}}_{\mathrm{t}\ \mathrm{Control}\ \mathrm{gene}}\right) $$

Gene expression fold (Test gene/Control gene) = 2^−^^△^^△Ct^.

### Total soluble sugar and proline content assay

In vitro plantlets treated in the same manner as for gene expression analysis were sampled for total soluble sugar and proline content assay under different stress conditions. The total soluble sugar content in the leaves was measured by the method of Zhang et al. [44]. The total soluble sugar was extracted from 100 mg potato leaves in boiling water and the supernatant was mixed with 80% ethanol to a final volume of 25 ml. Then, 2 mL of solution was mixed with 5 mL of anthrone reagent and the mixture was incubated at room temperature (18–30 °C) for 15 min. The absorbance was at 630 nm (UV-2600, Shomadzu) and the sugar concentration was determined from a glucose standard curve and expressed on a fresh weight base.

Proline content was assayed by the acid-ninhydrin procedure [45]. Potato leaf samples (0.5 g) were ground with 3% sulphosalicylic acid (10 mL). After centrifugation at 5000 rpm for 10 min, supernatant (2 mL) was mixed with the same volume of acidninhydrin and acetic acid, the mixture was incubated at 100 °C for 1 h, and the reaction was stopped on ice. The mixture was extracted with 4 mL toluene and absorbance at 517 nm was determined (UV-2600, Shimadzu). The proline concentration was determined from a standard curve and expressed on a fresh weight base.

### Statistical analysis

Gene expression, total soluble sugar, and proline assays were performed with three replicates and were analyzed using the Statistical Package for Social Sciences for Windows (SPSS version 17.0, SPSS Inc., Chicago, USA). Mean comparisons were performed using Tukey’s multiple range test and the significance between different treatments was presented at the *P* < 0.05 probability level.

## Results

### Identification of StSnRK2 members in potato

A genome-wide search using *SnRK2* cDNA sequences of *Arabidopsis*, maize and rice as references to identify the eight *SnRK2* homologous sequences in potato genome (*Solanum tuberosum* group Phureja DM1–3). The gene-specific primers were designed to clone the eight *SnRK2s* homologous cDNA from the potato cultivar ‘Longshu 3*’*. The candidate sequences coding potato *SnRK2s* ranged from 1008 bp to 1089 bp (Table 1). The eight sequences were designated as *StSnRK2.1*, *StSnRK*2.2 to *StSnRK2.8* and the confirmed full length cDNA were submitted to NCBI. The length of the predicted coding sequences of potato *SnRK2s* ranged from 37.7 4 kDa to 41.5 kDa. The protein length varied from 335 (*StSnRK2.1*) to 362 amino acids (*StSnRK2.3*). The protein length and the molecular weight of *StSnRK2s* were similar to *SnRK2* members reported in *Arabidopsis*, rice and maize (Table 1, Additional file 2: Figure S1) [14].

**Table 1:** The basic properties and characteristics of StSnRK2 genes in potato

Gene	Chr^a^	NCBI GI^b^	Genomic locus^c^	Position^d^	Cds (bp)^e^	Exons (No)^f^	Full lengh (bp)^g^	Amino acid (aa)^h^	Theoretical pI^i^	Mw (kDa)^j^
*StSnRK*2.1	4	404,435,142	PGSC0003DMT400079214	56,031,065…56,034,492	1008	9	3428	335	5.37	37.74
*StSnRK*2.2	8	404,435,144	PGSC0003DMT400067424	37,772,908…37,775,887	1020	9	4028	339	5.93	38.33
*StSnRK*2.3	1	404,435,146	PGSC0003DMT400066617	77,595,506...77599207	1089	9	3702	362	4.96	41.06
*StSnRK*2.4	1	404,435,148	PGSC0003DMT400061170	73,495,432...73500028	1083	9	4597	360	5.52	41.49
*StSnRK*2.5	4	404,435,150	PGSC0003DMT400060762	6,332,105...6336032	1035	9	3928	344	5.76	39.20
*StSnRK*2.6	5	404,435,152	PGSC0003DMT400060263	46,960,739...46964637	1077	7	3899	358	6.08	41.20
*StSnRK*2.7	12	404,435,154	PGSC0003DMT400045810	53,346,801...53350990	1011	9	4190	336	5.57	38.40
*StSnRK*2.8	11	404,435,156	PGSC0003DMT400041671	4,225,503…4,229,063	1050	9	2561	349	4.88	39.85

^a^Chromosome in which target gene is located in potato. ^b^Genebank index number in NCBI. ^c^Genomic locus in potato Genome Sequencing Consortium database (http://potatogenome.net/index.php/Main_Page). ^d^The position of genes on the corresponding list in ‘Genomic locus’. ^e^Length of coding region in base pairs. ^f^The numbers of exons which were analyzed with splign (http://www.ncbi.nlm.nih.gov/sutils/splign). ^g^Nucleotide accession of full-length cDNA in potato Genome Sequencing Consortium database. ^h^Amino acid number of deduced protein. ^i^The Theoretical pI of deduced protein. ^j^The molecular weight of deduced protein.

Based on the potato genome sequence, *StSnRK*2 genes were located on different chromosomes (Table 1) with *StSnRK*2.1 and *StSnRK*2.5 on chromosome 4, *StSnRK*2.3 and *StSnRK*2.4 on chromosome 1, and *StSnRK*2.2, *StSnRK*2.6, *StSnRK*2.7 and *StSnRK*2.8 on chromosomes 8, 5, 12 and 11, respectively.

### Gene and protein structures of *StSnRK2s*

**Fig. 1:**
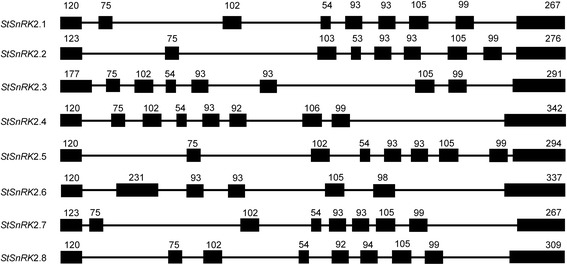
Gene structures of *StSnRK2s* in potato. Introns and exons were represented by lines and filled boxes, respectively. The numbers above the exons indicate the length (bp) of the exons

All *StSnRK*2s had nine exons except for *StSnRK*2.6 with seven exons (Fig. 1), and the first exon was around 120 bp except for *StSnRK2.3* with a 177 bp first exon. The seven genes with nine exons had highly conserved exon lengths, with the length of the second through the eighth exons 75 bp, 102 bp, 54 bp, 93 bp, 93 bp, 105 bp and 99 bp, respectively. *StSnRK2.6* had seven exons, and the length of the second exon showed a possible combination of the second to fourth conserved exons of the other *StSnRK2* genes. The predicted secondary structure of proteins suggested that eight StSnRK proteins might share a high degree of similarity (Table 2).

**Table 2:** The secondary structure of StSnRK2 protein sequences. The secondary structure of the deduced polypeptide was predicted using the programs of SOPMA which listed in Expasy (www.EXPASY.org)

Gene	Alpha helix (%)	Extended strand (%)	Beta turn (%)	Random coil (%)
*StSnRK*2.1	42.39	16.42	7.46	33.73
*StSnRK*2.2	43.36	16.22	6.78	33.63
*StSnRK*2.3	38.12	17.40	7.46	37.02
*StSnRK*2.4	47.22	15.56	9.17	28.06
*StSnRK*2.5	34.59	19.19	6.69	39.53
*StSnRK*2.6	43.02	13.41	5.31	38.27
*StSnRK*2.7	43.45	15.48	4.17	36.90
*StSnRK*2.8	38.68	16.62	7.16	37.54

### Phylogenetic analysis of StSnRK2

**Fig. 2:**
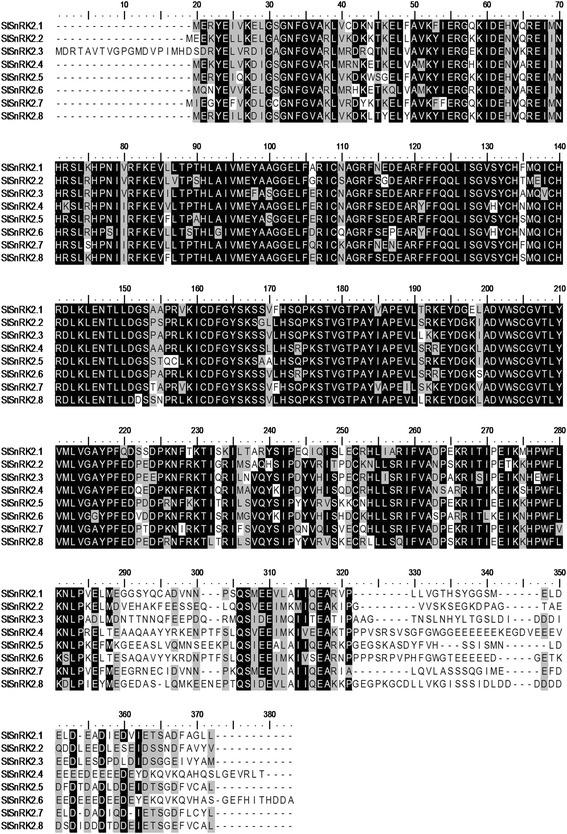
Alignment of the amino acid sequences of StSnRK2s. Identical amino acid residues are covered by black, similar residues are indicated by gray and the gaps in the sequences are indicated by dashes

**Fig. 3:**
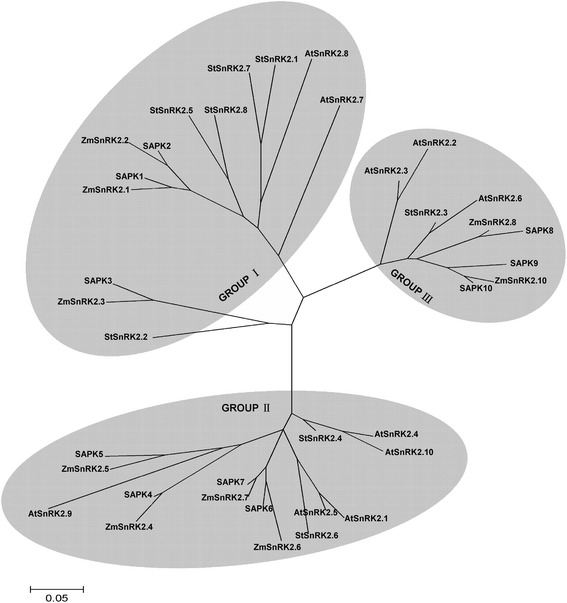
A phylogenetic tree constructed with CluxtalX1.8 using the SnRK2s full length amino acid sequence from potato, *Arabidopsis*, rice and maize. The bootstrap analysis was performed using 1000 replicates in MEGA (5.0) to evaluate the reliability of different phylogenetic groups

The N-termini of *StSnRK2s* were almost identical, but the C-terminal regions were highly divergent (ranging from 261 to 327 aa) (Fig. 2). All *StSnRK2s* had a conserved Ser/Thr protein kinase domain in the N-terminal (4 to 260 aa). Multiple alignment analysis based on the full length amino acid sequences of 37 *SnRK2s* was conducted to understand the evolutionary relationship between *StSnRK2s* and other reported *SnRK2s*. An unrooted phylogenetic tree was constructed with the amino acid sequences, using the neighbor-joining method (Fig. 3 and Additional file 2: Figure S2). The results demonstrated that *SnRK2s* are highly conserved in the plant kingdom. *StSnRK2s* were related to each other and could be divided into three distinct groups. The *StSnRK2.1, StSnRK2.2, StSnRK2.5, StSnRK2.7* and *StSnRK2.8* belonged to group I, *StSnRK2.4* and *StSnRK2.6* to group II and *StSnRK2.3* alone to group III.

### Subcellular localization of StSnRK2 proteins

**Fig. 4:**
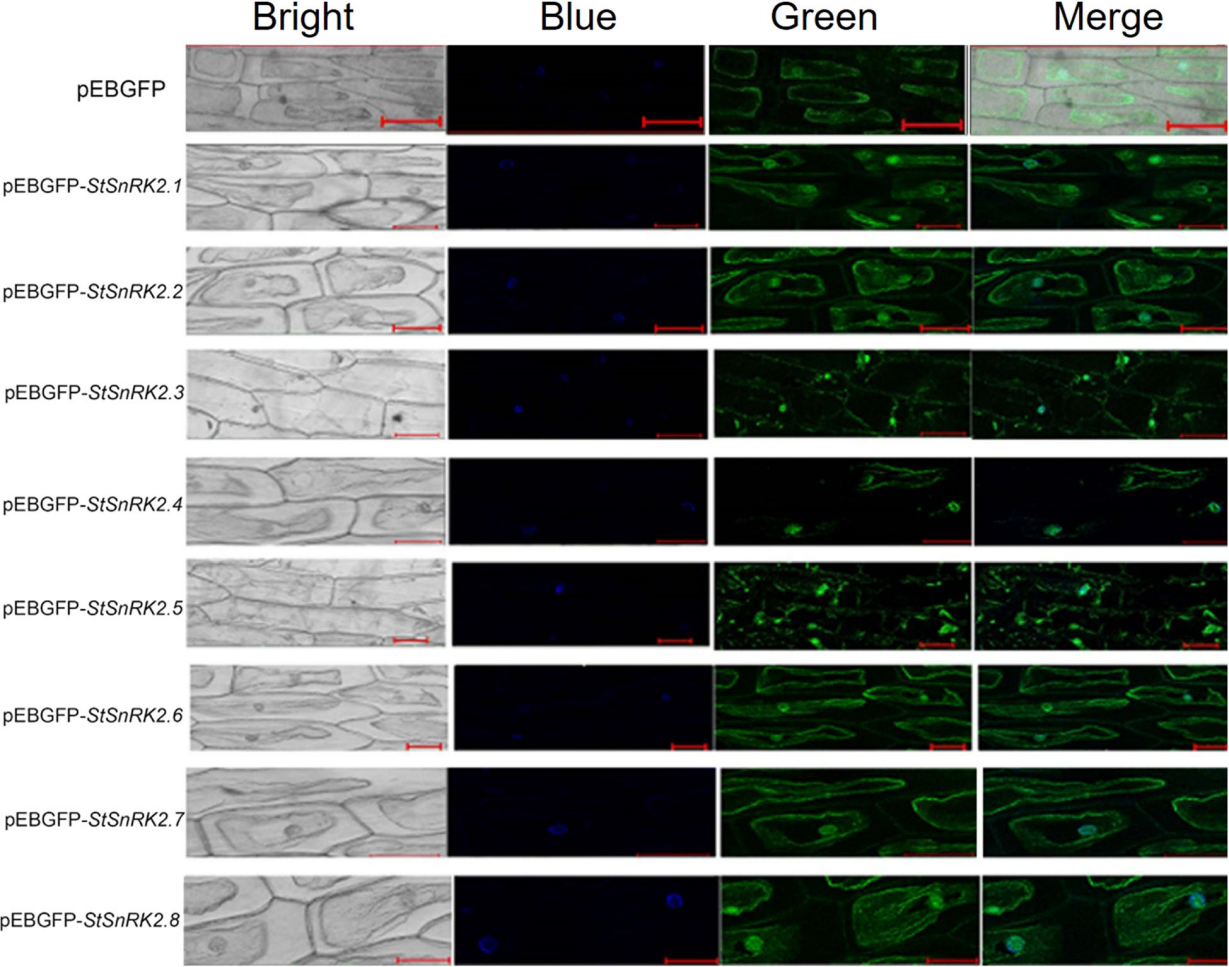
pBEGFP-StSnRK2 protein targeted to nucleus and cytoplasm in onion epidermal cells. The pEBGFP and pEBGFP-StSnRK2s plasmids were introduced into *Agrobacterium tumefaciens* strain EHA105 and were used for transformation into onion epidermal cells. Onion epidermal tissues were subsequently incubated on MS solid nutrient medium in the dark at 26 °C for 48 h. Localization of fluorescent proteins in onion epidermal cells were observed by a Zeiss LSM 710/ConfoCor2 laser-scanning imaging system (CarlZeiss, Jena, Germany). Fluorescence was detected between 505 and 550 nm with excitation at 488 nm. GFP fluorescence and light field vision were recorded in separate channels and then merged into an overlay image. Bar = 50 μm

To better understand the functions of StSnRK2, we transiently expressed pBEGFP-StSnRK2 fusion proteins in onion epidermal cells (Fig. 4). The epidermal cells were transformed with the 35S: GFP observed with fluorescence microscopy. Transiently expressed GFP-StSnRK2 fusion proteins were all detected in the nucleus or cytoplasm of onion epidermal cells. The fluorescence of GFP fused with *StSnRK2.1, StSnRK2.2*, *StSnRK2.6*, *StSnRK2.7,* and *StSnRK2.8* was detected in the nucleus and cytoplasm of onion epidermal cells. On the other hand, *StSnRK2.3* and *StSnRK2.4* are mainly associated to the nucleus, while *StSnRK2.5* seems to be associated to subcellular organelles.

### Tissue-specific expression of StSnRK2 genes

**Fig. 5:**
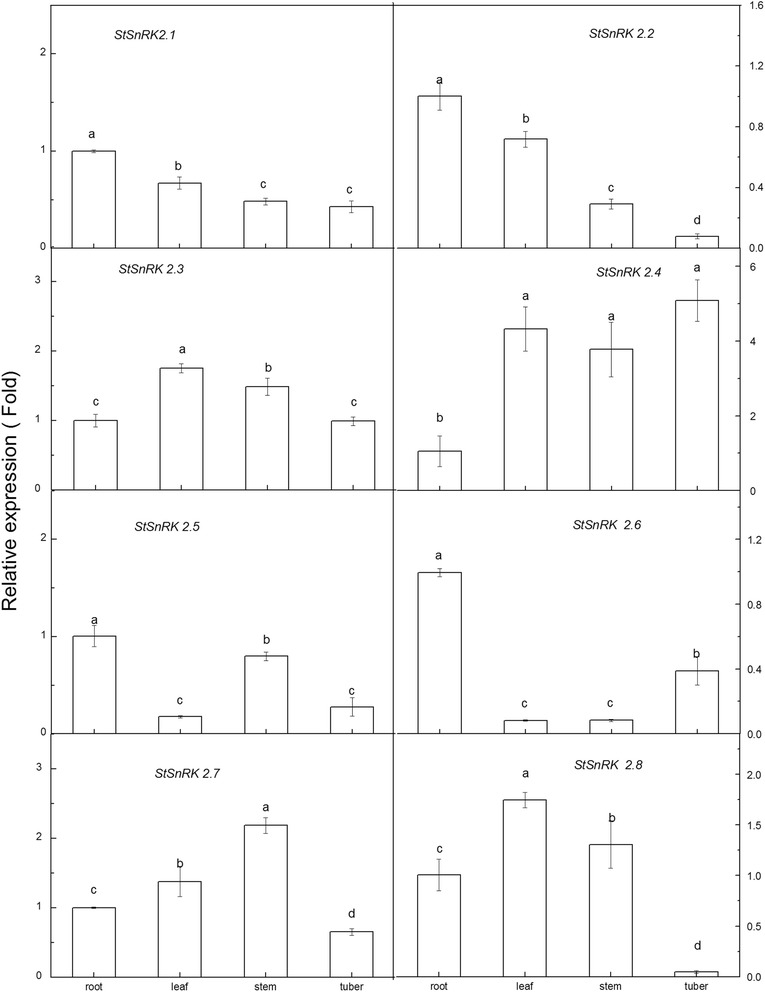
Results are presented as differential relative transcript abundance of *StSnRK*2.1 to *StSnRK*2.8 in different tissues; data represent the means ± SD of three replicates and different letters indicate significant difference at *P* < 0.05. Total RNA was extracted from 60-day-old plants. Y-axis showed the transcript fold to that in roots

The relative expressions of the eight *StSnRK2* genes varied among tissues (root, leaf, stem and tuber) (Fig. 5). *StSnRK2.1*, *StSnRK2.2*, *StSnRK2.5* and *StSnRK2.6* were highest expressed in root. In contrast, the expression levels of *StSnRK2.3*, *StSnRK 2.7* and *StSnRK 2.8* in leaf and stem were significantly higher than those in root (*P* < 0.05). *StSnRK2.4* expression in leaf, stem and tuber were significantly higher than that in root. The expressions of *StSnRK2.1*, *StSnRK2.2*, *StSnRK2.5*, *StSnRK2.6*, *StSnRK2.7* and *StSnRK2.8* were lower in tubers than those in root.

### Stress-induced expression of StSnRK2s and physiological responses of potato plants

**Fig. 6:**
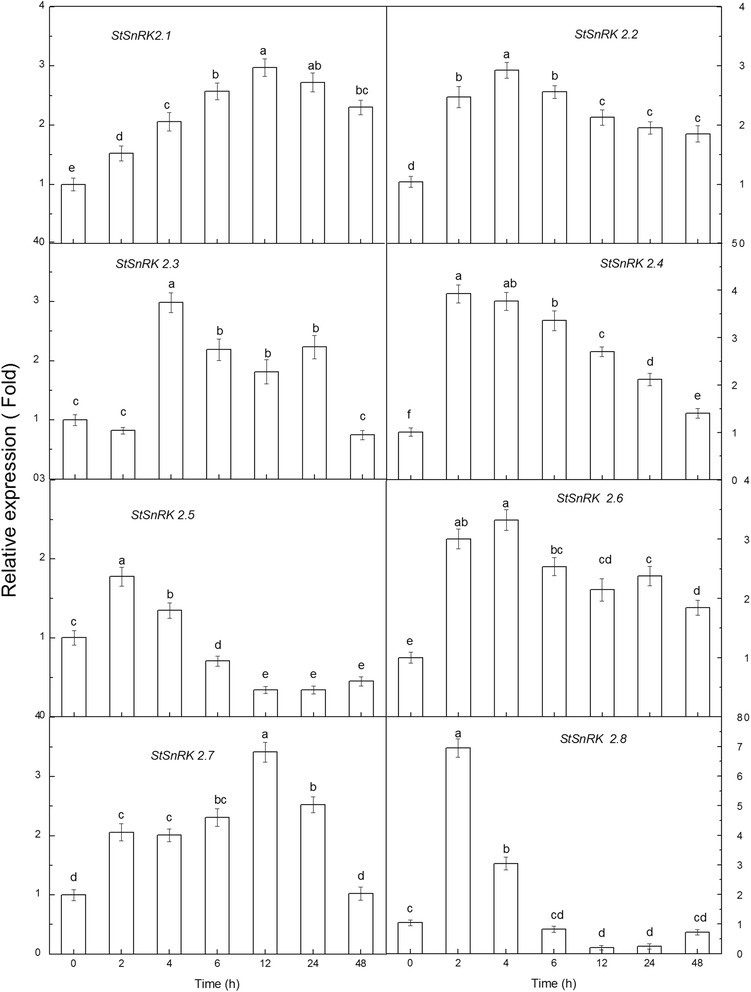
The differential relative transcript expression level of *StSnRK*2.1 to *StSnRK*2.8 under 200 mM NaCl stress treatments; data represent the means ± SD of three replicates and different letters indicate significant difference at *P* < 0.05. Total RNA was extracted from four-week-old plants subjected to 200 mM NaCl stress treatments for 2–48 h. Y-axis showed the transcript fold to that in the CK

Potato plantlets were treated with H_2_O (Control), ABA (50 μM), NaCl (200 mM) and PEG-6000 (5%). The total soluble sugar and proline contents were analyzed after 0 h, 2 h, 4 h, 6 h, 12 h, 24 h and 48 h. The results indicated that ABA (50 μM), NaCl (200 mM) and PEG-6000 (5%) treatments increased the total soluble sugar and proline with prolonging of treatments (Additional file 2: Figure S3). The relative gene expression of *StSnRKs* was analyzed using qRT-PCR. The results showed that the expression of all genes rose firstly after NaCl treatment, but different genes showed different responses to the stress condition (Fig. 6). *StSnRK2.1* and *StSnRK2.7* were similar and had their relative expression levels were highest at 12 h after subjected to the stress. Their expression levels gradually declined after 12 h, but *StSnRK2.1* had less reduction, and was significantly higher at 48 h, while *StSnRK2.7* declined greatest. The *StSnRK2.2*, *StSnRK2.4* and *StSnRK2.6* had similar expression trends. The relative expression levels of *StSnRK2.5* and *StSnRK2.8* increased rapidly after 2 h of treatments, and then began to fall to the levels lower than the control after the 6 h, and remained at a relatively low expression. The relative expression level of *StSnRK2.3* increased significantly 4 h after the stress, then began to decrease, but remained significantly high from 6 to 24 h and fell to the control level after 48 h of treatments, possibly indicating they have various signaling pathways.

**Fig. 7:**
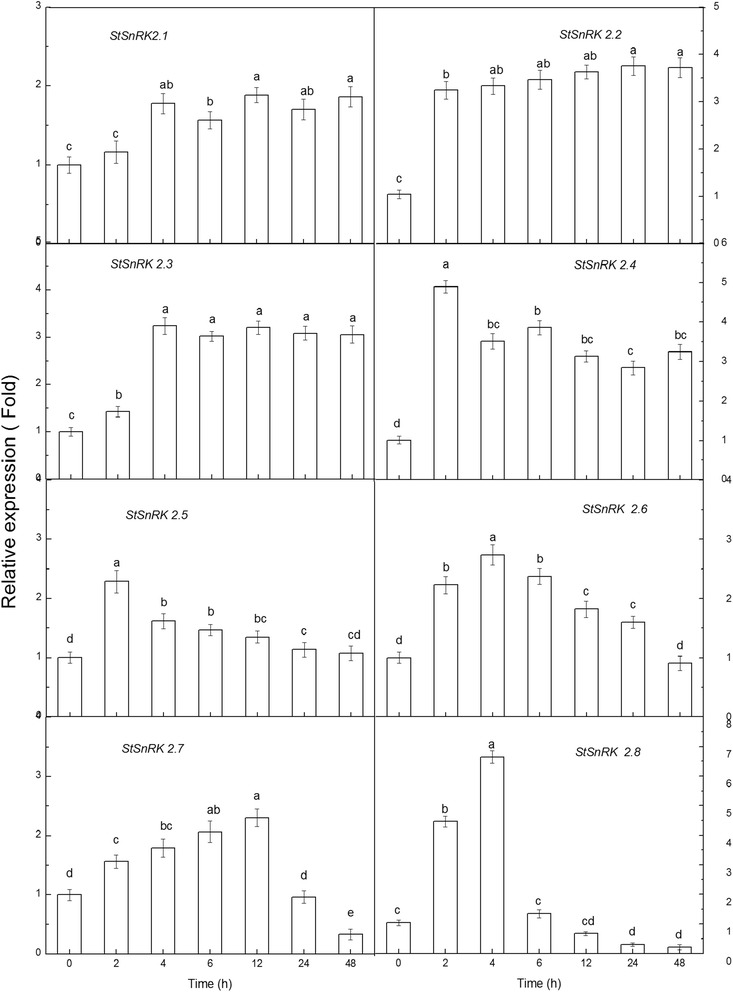
The differential relative transcript expression level of *StSnRK*2.1 to *StSnRK*2.8 under 5% PEG stress treatments; data represent the means ± SD of three replicates and different letters indicate significant difference at *P* < 0.05. Total RNA was extracted from four-week-old plants subjected to 5% PEG stress treatments for 2–48 h. Y-axis showed the transcript fold to that in the CK

Under PEG (5%) treatment, the relative expression of *StSnRK2.1*, *StSnRK2.2* and *StSnRK2.3* rapidly rose after 2 h–4 h of treatment, and remained a relative stable level until 48 h (Fig. 7). *StSnRK2.1* relative expression increased significantly after 4 h, but the relative expression levels of *StSnRK2.2* and *StSnRK2.3* increased rapidly after 2 h of treatment. The relative expression levels of *StSnRK2.4* and *StSnRK2.5* rose rapidly, about five times of the control and reached a peak at 2 h of treatment, then declined significantly. The relative expression of *StSnRK2.6* declined slowly with the prolonging of treatment time; however, the relative expression of *StSnRK2.8* was about seven times of the control at 4 h of treatment and then declined after 6 h of exposure to stress. *StSnRK2.7* expression slowly increased with time until 12 h of treatment, then decreased significantly with no obvious difference compared to control at 24 h of treatment and to less than half of the control at 48 h of treatment.

**Fig. 8:**
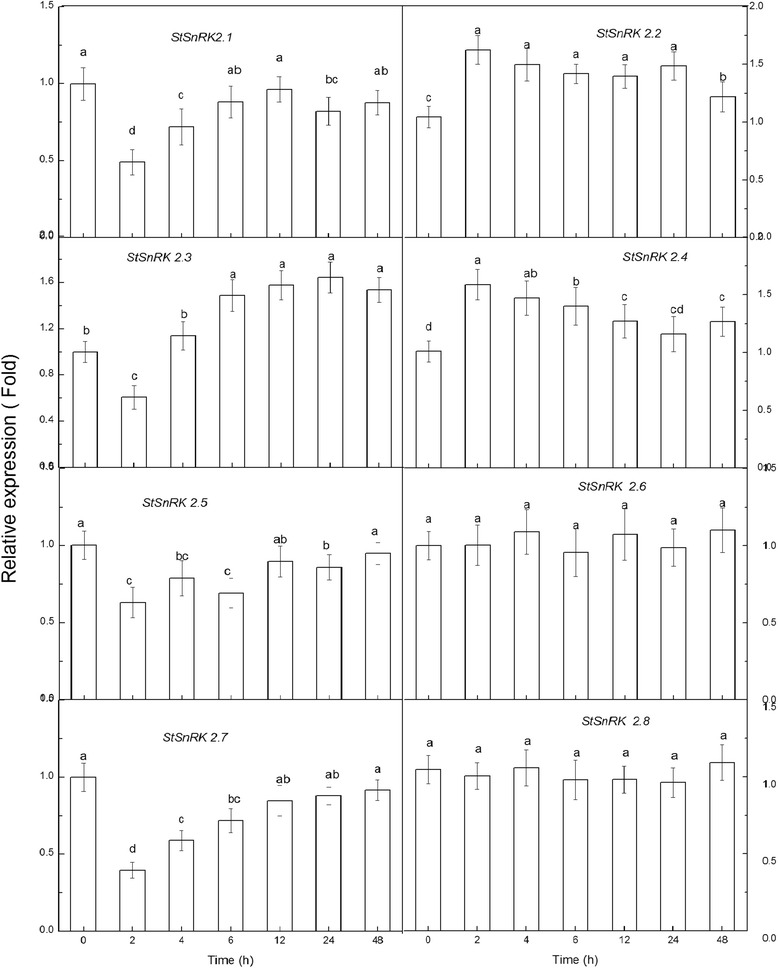
The differential relative transcript expression level of *StSnRK*2.1 to *StSnRK*2.8 under 50 μM ABA stress treatments, the data represent the means ± SD of three replicates and different letters indicate significant difference at *P* < 0.05. Total RNA was extracted from four--week-old plants subjected to 50 μM ABA stress treatments for 2–48 h. Y-axis showed the transcript fold to that in the CK

The relative expression pattern of *StSnRK2* genes under ABA treatment were different from NaCl and PEG treatments (Fig. 8). After 2 h of ABA, the relative expression level of *StSnRK2.3* declined slightly, increased to a maximum after 6 h and then remained stable. Other members had no obvious changes in expression under ABA treatment, although the expression levels of *StSnRK2.1*, *StSnRK2.5* and *StSnRK2.7* declined slightly at 2 h, but gradually returned to the control levels after 4 h of treatment. Compared to NaCl and PEG treatments, there was no significantly increasing trend. After 2 h of treatment, the expression levels of *StSnRK2.2* and *StSnRK2.4* also increased, but those of *StSnRK2.6* and *StSnRK2.8* had not changed. The results showed that *StSnRK2* genes have no obvious response to ABA; most members of the subfamily may be involved in plant response pathways to stresses and were ABA-independent except that *StSnRK2.3* is ABA-dependent.

To identify the presence of cis-elements in the promoter regions of the *StSnRK*2s, the 2 kb length of the upstream region was considered as the promoter region and used to search for stress-responsive elements. We cloned eight StSnRK2 genes from the potato cultivar ‘Longshu-3’ and analyzed their location in the genome, gene structure, amino acid sequence divergence and tissue specific and stress-induced expression patterns. All genes except for *StSnRK2.4* carried ABRE, *StSnRK2.1*, *StSnRK2.2* and *StSnRK2.4* carried DRE/CRT and StSnRK2.1, *StSnRK2.2, StSnRK2.4*, *StSnRK2.5* and *StSnRK2.7* had LTRE.

## Discussions

The *SnRK2* subfamily is an osmotic-stress activated protein kinase and has been identified and characterized in *Arabidopsis*, rice, maize and other plant species. Many studies have showed that SnRK2 members have potential roles in improving stress tolerance and increase crop yields [29, 32]. In the present study, we identified and cloned eight *StSnRK2* genes from potato cultivar ‘Longshu-3’, and analyzed their genome distribution, gene structure, phylogenic and tissue specific and stress-induced expression patterns. Similar to *SnRK2s* in *Arabidopsis*, rice and maize, *StSnRK*2 genes in potato were distributed over several chromosomes. All the *StSnRK2s* except for *StSnRK2.6* have nine exons, similar to the *SnRK2s* in other plant species [26, 46]. The functional characteristics of the C-terminal region of *SnRK2s* has been reported in several plant species like *Arabidopsis* [4, 5, 13, 14, 16, 17, 35], rice [25, 36] and maize [26, 47]. The evolutionary distance of *StSnRK2.3* is very close to *AtSnRK2.6* (Fig. 3). The gene products of *AtSnRK2.2* and AtSnRK2.3 are mostly concentrated in leaves, contributing largely to the ABA-dependent stomatal regulation [4, 15].

Tissue-specific expression of *SnRK2* genes were observed in many species. For example, fava bean AAPKs induced by ABA signaling were mainly expressed in guard cells [48]. The expression of *SAPK* members is different among tissues in rice: *SAPK8, 9* and *10* are regulated only by ABA in blades and roots, with the level of expression in roots higher than in other tissues [25]. Root tips, as a dynamic and specialized tissue, play a crucial role in sensing water and nutrients and often have rapid responses by transmitting appropriate signals. In current study, the expressions of *StSnRK*2*s* in various tissues of potato plantlets were highly diversified and these proteins may mediate various metabolic processes under normal growing conditions. The expression of *StSnRK2.4* was higher in shoot tissues than in roots, and could be induced by NaCl, ABA and PEG (Figs. 5, 6, 7, 8), suggesting that *StSnRK2.4* may be sensitive to stress signals and function in osmotic-stress responses in shoot tissues of potato. The expression of *TaSnRK2.4* was stimulated by high salt treatments, and the overexpression of *TaSnRK2.4* in *Arabidopsis* enhanced salt tolerance [28]. *StSnRK2.4* and *AtSnRK2.4* were classified into the same group, implying that *StSnRK2.4* may be a potential target for improving the salt tolerance of potato. In *Arabidopsis*, *SnRK2.2*, *SnRK2.3* and *SnRK2.6* are typically activated by ABA, and can phosphorylate the ABA-responsive elements. So, they are important for the activation of ABA-responsive genes. In rice, *SAPK8, 9,* and *10* are activated by ABA [25]. The regulation of the plant responses to ABA via SnRK2s pathways occurs by direct phosphorylation of various downstream targets, for example, SLAC1, KAT1, AtRbohF and transcription factors were required for the expression of numerous stress response genes [46]. The expression of *ZmSnRK2* genes can be induced by various stress treatments, suggesting their potential roles in stress responses. We found that each gene of *StSnRK2* subfamily has a unique expression pattern in different tissues of potato, with the expressions of *StSnRK2.2* and *StSnRK2.8* very low in roots, whereas the expressions of *StSnRK2.5* and *StSnRK2.6* were very low in leaves. However, they were insensitive to ABA treatments, probably due to the different regulatory elements that each *StSnRK2* carried on the 2-kb upstream region (Table 3). Alternatively, the concentration of ABA and the prolonging of the ABA treatment need to be further discussed. The manipulation of *StSnRK2s* can be a valuable approach for improving the stress tolerance of potato.

**Table 3:** Putative Cis elements existed in the 2 kb upstream region of StSnRK2 genes

Name	ABRE	DRE/CRT	LTRE
*StSnRK*2.1	4	1	1
*StSnRK*2.2	3	2	1
*StSnRK*2.3	1	0	0
*StSnRK*2.4	0	2	2
*StSnRK*2.5	2	0	2
*StSnRK*2.6	2	0	0
*StSnRK*2.7	3	0	1
*StSnRK*2.8	5	0	0

The sequence of ABRE elements include ACGTG, MACGYGB, TACGTGTC, YACGTGGC, and CCACGTGG. The sequence of DRE/CRT element include RCCGAC, ACCGAC, ACCGAGA and GTCGAC. The sequences of LTRE element include CCGAC, CCGAAA, ACCGACA and CCGAC

## Conclusions

In the present study, we identified and characterized eight *SnRK2* genes in the potato genome. The eight *StSnRK2s* exhibit similar gene structure and secondary structures in potato to the *SnRK2s* found in other plant species. The fluorescence of GFP fused with *StSnRK2.1, StSnRK2.2*, *StSnRK2.6*, *StSnRK2.7,* and *StSnRK2.8* was detected in the nucleus and cytoplasm of onion epidermal cells. On the other hand, *StSnRK2.3* and *StSnRK2.4* are mainly associated to the nucleus, while *StSnRK2.5* seems to be associated to subcellular organelles. The relative expression of the eight *SnRK2* genes varied among tissues (roots, leaves, tubers and stems) and abiotic stresses (ABA, NaCl and PEG-6000) with the prolonging of treatments. This study provides valuable information for the future functional dissection of potato *SnRK2* genes in stress signal transduction, plant growth and development studies.

## Additional files

Additional file 1: **Table S1** and **Table S2**. **Table S1**. The Accession numbers of *SnRK*2s from *Arabidopsis*, rice and maize. **Table S2**. Oligonucleotides used for *StSnRK*2 cloning and qRT-PCR. (DOCX 21 kb)

Additional file 2: **Figure S1**, **Figure S2**﻿ and **﻿Figure S3**. **Figure S1**. Alignment of the amino acid sequences of the *SnRK2s* from *Arabidopsis*, rice, maize and potato. Identical amino acids residues are covered by black, similar residues are indicated by gray, Dashes indicate gaps in the sequences to allow maximal alignment. **Figure S2**. The polygenetic tree was constructed with (CluxtalX1.8) using the *SnRK2s* full length amino acid sequence from potato. The bootstrap values are in percentage. **Figure S3**. The proline and total soluble sugar content under 200 mM NaCl, 5% PEG, and 50 μM ABA treatments. Data represent the means ± SD of three replicates and different letters indicate significant difference at *P* < 0.05. (DOCX 1796 kb).

## Abbreviations

ABA: Abscisic acid
NaCl: Sodium chloride
PEG-6000: Polyethyleneglycol-6000
qRT-PCR: Quantitative real-time PCR
SnRK: Sucrose non-ferment 1 related protein kinase
