# Supplementary material for: Sucrose non-ferment 1 related protein kinase 2 (SnRK2) genes could mediate the stress responses in potato (Solanum tuberosum L.)
Source: BMC Genet. 2017 May 15;18:41. doi: 10.1186/s12863-017-0506-6 (PMC5433004; doi:10.1186/s12863-017-0506-6)
Supplement: Supplementary file 1 — Table S1 and Table S2. Table S1. The Accession numbers of SnRK2s from Arabidopsis, rice and maize. Table S2. Oligonucleotides used for StSnRK2 cloning and qRT-PCR. (DOCX 21 kb) [file 12863_2017_506_MOESM1_ESM.docx]

**Table S1** The Accession numbers of *SnRK2s* from Arabidopsis, rice and maize

| Genes | Accession No. | organism | Reference |
| --- | --- | --- | --- |
| SAPK1 | AB125302 | *Oryza sativa Japonica* | (Kobayashi,Y.,2004) |
| SAPK2 | AB125303 | [*Oryza sativa Japonica*](http://www.ncbi.nlm.nih.gov/Taxonomy/Browser/wwwtax.cgi?id=39947) | (Kobayashi,Y.,2004) |
| SAPK3 | AB125304 | [*Oryza sativa Japonica*](http://www.ncbi.nlm.nih.gov/Taxonomy/Browser/wwwtax.cgi?id=39947) | (Kobayashi,Y.,2004) |
| SAPK4 | AB125305 | [*Oryza sativa Japonica*](http://www.ncbi.nlm.nih.gov/Taxonomy/Browser/wwwtax.cgi?id=39947) | (Kobayashi,Y.,2004) |
| SAPK5 | AB125306 | [*Oryza sativa Japonica*](http://www.ncbi.nlm.nih.gov/Taxonomy/Browser/wwwtax.cgi?id=39947) | (Kobayashi,Y.,2004) |
| SAPK6 | AB125307 | [*Oryza sativa Japonica*](http://www.ncbi.nlm.nih.gov/Taxonomy/Browser/wwwtax.cgi?id=39947) | (Kobayashi,Y.,2004) |
| SAPK7 | AB125308 | [*Oryza sativa Japonica*](http://www.ncbi.nlm.nih.gov/Taxonomy/Browser/wwwtax.cgi?id=39947) | (Kobayashi,Y.,2004) |
| SAPK8 | AB125309 | [*Oryza sativa Japonica*](http://www.ncbi.nlm.nih.gov/Taxonomy/Browser/wwwtax.cgi?id=39947) | (Kobayashi,Y.,2004) |
| SAPK9 | AB125310 | [*Oryza sativa Japonica*](http://www.ncbi.nlm.nih.gov/Taxonomy/Browser/wwwtax.cgi?id=39947) | (Kobayashi,Y.,2004) |
| SAPK10 | AB125311 | [*Oryza sativa Japonica*](http://www.ncbi.nlm.nih.gov/Taxonomy/Browser/wwwtax.cgi?id=39947) | (Kobayashi,Y.,2004) |
| AtSnRK2.1 | NM_120946.4 | [*Arabidopsis thaliana*](http://www.ncbi.nlm.nih.gov/Taxonomy/Browser/wwwtax.cgi?id=3702) | (Swarbreck,D.,2011) |
| AtSnRK2.2 | NM_001203118.1 | [*Arabidopsis thaliana*](http://www.ncbi.nlm.nih.gov/Taxonomy/Browser/wwwtax.cgi?id=3702) | (Swarbreck,D.,2011) |
| AtSnPK2.3 | NM_126087.4 | [*Arabidopsis thaliana*](http://www.ncbi.nlm.nih.gov/Taxonomy/Browser/wwwtax.cgi?id=3702) | (Swarbreck,D.,2011) |
| AtSnPK2.4 | NM_001035944.2 | [*Arabidopsis thaliana*](http://www.ncbi.nlm.nih.gov/Taxonomy/Browser/wwwtax.cgi?id=3702) | (Swarbreck,D.,2011) |
| AtSnPK2.5 | NM_125760.1 | [*Arabidopsis thaliana*](http://www.ncbi.nlm.nih.gov/Taxonomy/Browser/wwwtax.cgi?id=3702) | (Swarbreck,D.,2011) |
| AtSnPK2.6 | NM_001125639.1 | [*Arabidopsis thaliana*](http://www.ncbi.nlm.nih.gov/Taxonomy/Browser/wwwtax.cgi?id=3702) | (Swarbreck,D.,2011) |
| AtSnPK2.7 | NM_120165.1 | [*Arabidopsis thaliana*](http://www.ncbi.nlm.nih.gov/Taxonomy/Browser/wwwtax.cgi?id=3702) | (Swarbreck,D.,2011) |
| AtSnPK2.8 | NM_202441 | [*Arabidopsis thaliana*](http://www.ncbi.nlm.nih.gov/Taxonomy/Browser/wwwtax.cgi?id=3702) | (Swarbreck,D.,2011) |
| AtSnPK2.9 | NM_127867.4 | [*Arabidopsis thaliana*](http://www.ncbi.nlm.nih.gov/Taxonomy/Browser/wwwtax.cgi?id=3702) | ( Lin,X.,2011) |
| AtSnPK2.10 | NM_104774.5 | [*Arabidopsis thaliana*](http://www.ncbi.nlm.nih.gov/Taxonomy/Browser/wwwtax.cgi?id=3702) | (Swarbreck,D.,2011) |
| ZmSnRK2.1 | EU676033 | [*Zea mays*](http://www.ncbi.nlm.nih.gov/Taxonomy/Browser/wwwtax.cgi?id=4577) | (Huai,J.,2008) |
| ZmSnRK2.2 | EU676034 | [*Zea mays*](http://www.ncbi.nlm.nih.gov/Taxonomy/Browser/wwwtax.cgi?id=4577) | (Huai,J.,2008) |
| ZmSnRK2.3 | EU676035 | [*Zea mays*](http://www.ncbi.nlm.nih.gov/Taxonomy/Browser/wwwtax.cgi?id=4577) | (Huai,J.,2008) |
| ZmSnRK2.4 | EU676036 | [*Zea mays*](http://www.ncbi.nlm.nih.gov/Taxonomy/Browser/wwwtax.cgi?id=4577) | (Huai,J.,2008) |
| ZmSnRK2.5 | EU676037 | [*Zea mays*](http://www.ncbi.nlm.nih.gov/Taxonomy/Browser/wwwtax.cgi?id=4577) | (Huai,J.,2008) |
| ZmSnRK2.6 | EU676038.1 | [*Zea mays*](http://www.ncbi.nlm.nih.gov/Taxonomy/Browser/wwwtax.cgi?id=4577) | (Huai,J.,2008) |
| ZmSnRK2.7 | EU676039 | [*Zea mays*](http://www.ncbi.nlm.nih.gov/Taxonomy/Browser/wwwtax.cgi?id=4577) | (Huai,J.,2008) |
| ZmSnRK2.8 | EU676040 | [*Zea mays*](http://www.ncbi.nlm.nih.gov/Taxonomy/Browser/wwwtax.cgi?id=4577) | (Huai,J.,2008) |
| ZmSnRK2.10 | EU676041 | [*Zea mays*](http://www.ncbi.nlm.nih.gov/Taxonomy/Browser/wwwtax.cgi?id=4577) | (Huai,J.,2008) |

**Table S2** Oligonucleotides used for *StSnRK2* cloning and qRT-PCR.

| Gene |  | Sequence of oligonucleotides forward (F) and reverse (R) | Gene Length(bp) |
| --- | --- | --- | --- |
| Primers for PCR cloning | | | |
| StSnRK2.1 | F | 5'-GCTCTAGA^a^ATGGAGCGTTATGAGATAGTGAAG-3' | 1008 |
|  | R | 5'-GCGAGCTC^b^TCACAGTAAACCAGCAAA ATCAG-3' |  |
| StSnRK2.2 | F | 5'-GCTCTAGA^a^ATGGAGGAAAAGTATGAGCTTTTG-3' | 1020 |
|  | R | 5'-GCGAGCTC^b^TCAGACATA AACAGCAAAGTCATTG-3' |  |
| StSnRK2.3 | F | 5'-GCTCTAGA^a^ATGGATCGGACGGCAGTGAC-3' | 1089 |
|  | R | 5'-GCGAGCTC^b^TTACATTGCATAGACAATCTCTCCAC-3' |  |
| StSnRK2.4 | F | 5'-GCTCTAGA^a^ATGGAGAAATACGAGCTTGTGAA-3' | 1083 |
|  | R | 5'-GCGAGCTC^b^TTAGGTGAGACGAACTTCCCC-3' |  |
| StSnRK2.5 | F | 5'-GCTCT AGA^a^ ATGGAA AGATATGAA ATTCA-3' | 1035 |
|  | R | 5'- GCGAGCTC^b^TCATAATGCACATACAA-3' |  |
| StSnRK2.6 | F | 5'-GCTCTAGA^a^ATGCAGAATTACGAAGTTGTGAAG-3' | 1077 |
|  | R | 5'-GCGAGCTC^b^TTAAGCATCATCATGTGTGATATGA-3' |  |
| StSnRK2.7 | F | 5'-GC TCTAGA^a^ATGATAGAGGGTTATGAGTTTGTG-3' | 1011 |
|  | R | 5'-GC GAGCTC^b^TCACAAATAACAAAGAAAGTCACC-3’ |  |
| StSnRK2.8 | F | 5'-GCTCTAGA^a^ATGGAAAGGTATGAAATTTTGAAAGA-3' | 1050 |
|  | R | 5'-GCGAGCTC^b^TCATAAAGCACAAACAAATTCCC-3' |  |
| Primers for qRT-PCR | | | |
| StSnRK2.1 | F | 5'- CAATGTGCCGATGTAAATAACCC-3' | 102 |
|  | R | 5'- CCCATAAGAATGTGCTCCAACC-3' |  |
| StSnRK2.2 | F | 5'-TACCTGGAGTAGTGTCA-3' | 106 |
|  | R | 5'-AAGTCATTGCTGCTGTC-3’ |  |
| StSnRK2.3 | F | 5'-ACCAGTTTGAGGAGCCA-3' | 175 |
|  | R | 5'-ATATCGAGGTCAGGATC-3' |  |
| StSnRK2.4 | F | 5'-TTCCCGTTCAGTATCAG-3' | 100 |
|  | R | 5'-TTCTTCATCCTCCTCCT-3' |  |
| StSnRK2.5 | F | 5'- ATAGTGAAGAAAAGCCATTACAAAG-3' | 162 |
|  | R | 5'- TCTCATCATCTAAATCAGCATCAGT-3' |  |
| StSnRK2.6 | F | 5'-GACAATCCAACATTCTCCCTTCA-3' | 81 |
|  | R | 5'- TGATGGAGGTGGTGGATTTCTT-3' |  |
| StSnRK2.7 | F | 5'- GGAAGAAGGACGGAATGAGTG-3' | 123 |
|  | R | 5'- ATGCCTTGAGATGAACTTGCT-3' |  |
| StSnRK2.8 | F | 5'-GGCCCAAAGGTTGTGAT-3' | 125 |
|  | R | 5'-AAATTCCCCACTTGTCT-3' |  |

a & b donate the restriction sites of *Xbal*I and *Sac*I, respectively
