# Supplementary material for: Sucrose non-ferment 1 related protein kinase 2 (SnRK2) genes could mediate the stress responses in potato (Solanum tuberosum L.)
Source: BMC Genet. 2017 May 15;18:41. doi: 10.1186/s12863-017-0506-6 (PMC5433004; doi:10.1186/s12863-017-0506-6)
Supplement: Supplementary file 2 — Figure S1, Figure S2﻿ and ﻿Figure S3. Figure S1. Alignment of the amino acid sequences of the SnRK2s from Arabidopsis, rice, maize and potato. Identical amino acids residues are covered by black, similar residues are indicated by gray, Dashes indicate gaps in the sequences to allow maximal alignment. Figure S2. The polygenetic tree was constructed with (CluxtalX1.8) using the SnRK2s full length amino acid sequence from potato. The bootstrap values are in percentage. Figure S3. The proline and total soluble sugar content under 200 mM NaCl, 5% PEG, and 50 μM ABA treatments. Data represent the means ± SD of three replicates and different letters indicate significant difference at P < 0.05. (DOCX 1796 kb). [file 12863_2017_506_MOESM2_ESM.docx]

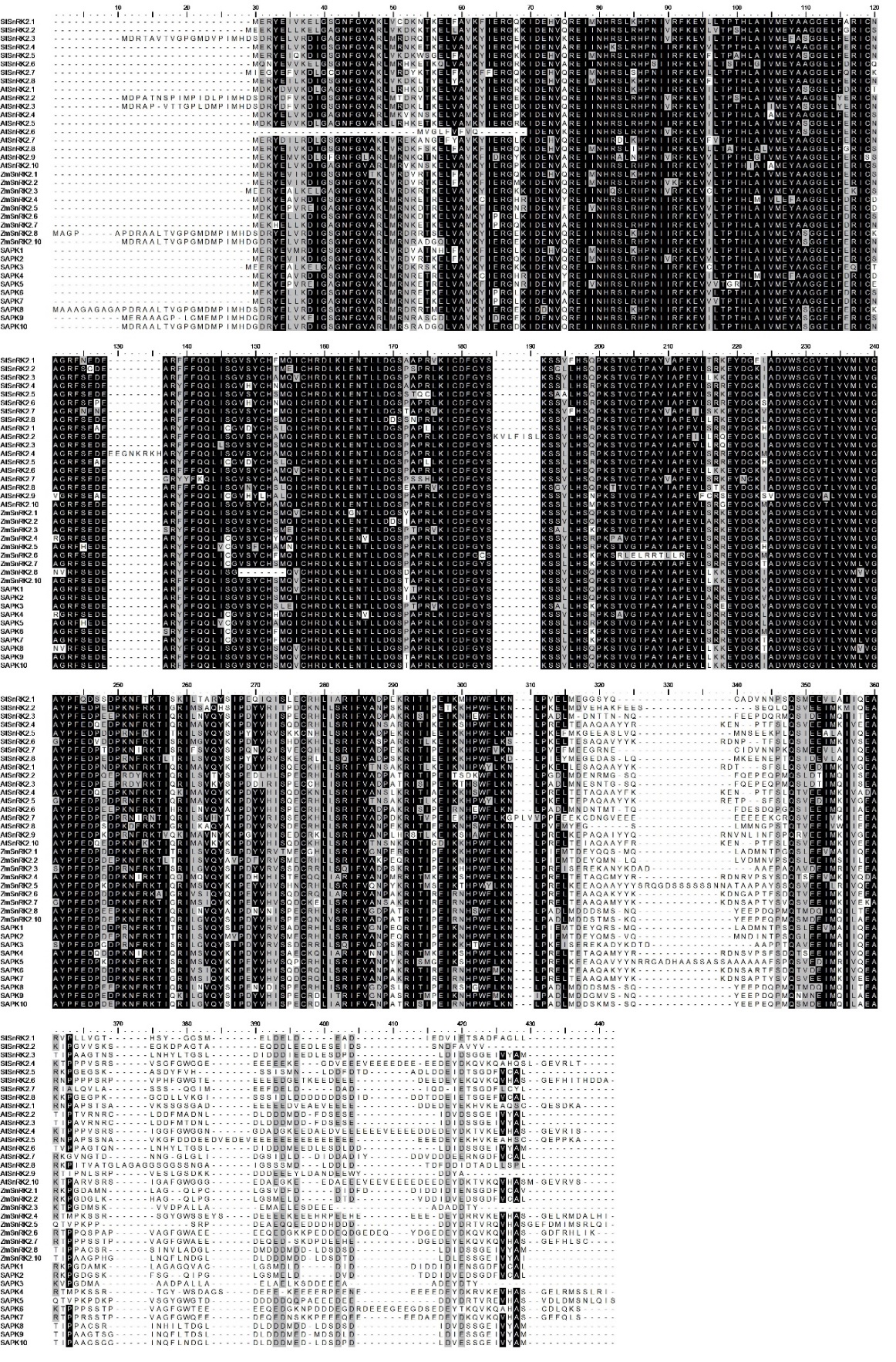


**Fig. S1** Alignment of the amino acid sequences of the *SnRK2s* from *Arabidopsis*, rice, maize and potato. Identical amino acids residues are covered by black, similar residues are indicated by gray, Dashes indicate gaps in the sequences to allow maximal alignment.


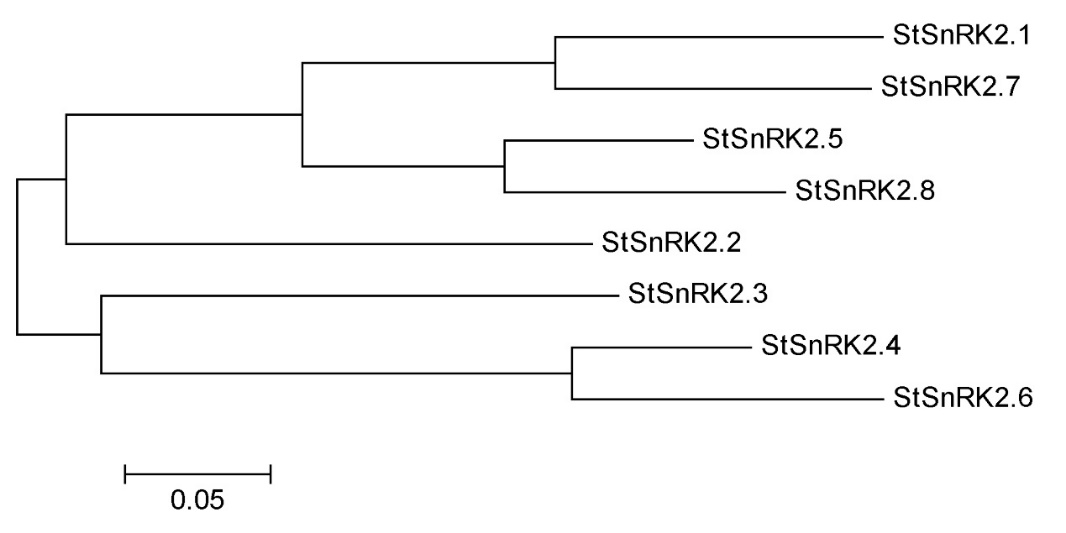


**Fig. S2** The polygenetic tree was constructed with (CluxtalX1.8) using the *SnRK2s* full length amino acid sequence from potato. The bootstrap values are in percentage.


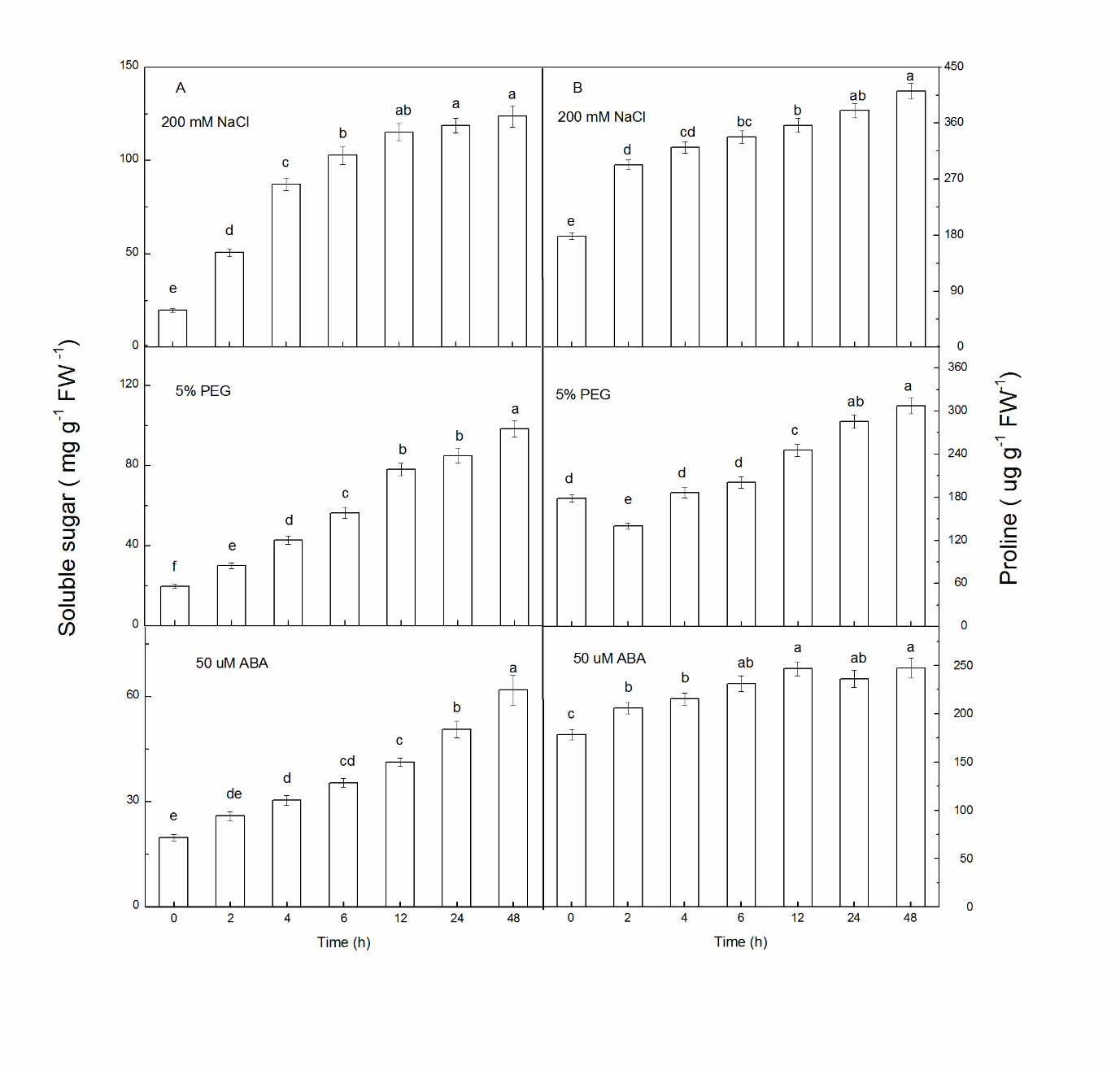


**Fig. S3** The proline and total soluble sugar content under 200mM NaCl, 5% PEG and 50µM ABA treatments. Data represent the means ± SD of three replicates and different letters indicate significant difference at *P* < 0.05.
